# Reinforcement Learning-Based Data Association for Multiple Target Tracking in Clutter

**DOI:** 10.3390/s20226595

**Published:** 2020-11-18

**Authors:** Chengzhi Qu, Yan Zhang, Xin Zhang, Yang Yang

**Affiliations:** School of Aeronautics and Astronautics, Sun Yat-sen University, Shenzhen 518000, China; quchzh@mail2.sysu.edu.cn (C.Q.); zhangx639@mail2.sysu.edu.cn (X.Z.); yiyinfeixiong@gmail.com (Y.Y.)

**Keywords:** data association, multiple target tracking, reinforcement learning, joint probabilistic data association

## Abstract

Data association is a crucial component of multiple target tracking, in which each measurement obtained by the sensor can be determined whether it belongs to the target. However, many methods reported in the literature may not be able to ensure the accuracy and low computational complexity during the association process, especially in the presence of dense clutters. In this paper, a novel data association method based on reinforcement learning (RL), i.e., the so-called RL-JPDA method, has been proposed for solving the aforementioned problem. In the presented method, the RL is leveraged to acquire available information of measurements. In addition, the motion characteristics of the targets are utilized to ensure the accuracy of the association results. Experiments are performed to compare the proposed method with the global nearest neighbor data association method, the joint probabilistic data association method, the fuzzy optimal membership data association method and the intuitionistic fuzzy joint probabilistic data association method. The results show that the proposed method yields a shorter execution time compared to other methods. Furthermore, it can obtain an effective and feasible estimation in the environment with dense clutters.

## 1. Introduction

Measurement data association in a cluttered environment is considered to be a high potential and challenging technique in the field of multiple target tracking [1,2]. The main mission of data association is that each measurement obtained by the sensor should be determined whether it belongs to the target when multiple targets are present [3,4]. However, clutters such as false alarms and electronic countermeasures make it very difficult to accomplish the data association mission efficiently. Therefore, many methods in the literature have been proposed to solve this problem [5,6,7]. The nearest neighbor data association method (NN) [8] selects a measurement that owns the shortest distance with the predicted measurement of the target in the association environment and complete the data association. However, the nearest measurement may be a clutter and the mission ultimately failed. Reference [9] proposed a fuzzy based nearest-neighbor association method for multiple targets tracking. Instead of the classical Mahalanobis distance, fuzzy clustering has been used to acquire a likelihood measure. The probabilistic data association (PDA) [10] method calculates the association probability between obtained measurements and target, which is only applicable in assigning multiple measurements to a single target. Reference [11] proposed a novel data association technique, which is made up of PDA and NN. The probability of each measurement is obtained from the conditional probability density functions of the interested events. A multiple hypothesis tracker (MHT) [12] has been proposed to evaluate the likelihood for tracking systems. A list that can be sorted by the probability estimates of hypotheses is considered as the outputs of MHT. However, all the possible association hypotheses attempt to be maintained in the MHT method over time, which means a high computational complexity. To track multiple targets in multiple-detection systems, reference [13] developed a multiple detection multiple hypothesis tracker (MD-MHT). During the extension to the multi-frame assignment method, the proposed method solves the data association problem effectively.

As the multi-target version of PDA, the joint probabilistic data association (JPDA) [14] method has stronger applicability. At each scan, infeasible measurements are eliminated using a gating judgment. Multiple joint events based on measurements are obtained, and the corresponding posterior probabilities are then computed. However, the probability calculation of joint events seems complicated, and the dimension explosion problem will occur in the calculation of the posterior probabilities with the increase of clutters and targets [15]. Despite many new methods, which have been proposed when dealing with the multiple targets tracking problem such as the probability hypothesis density filter (PHD) [16], the cardinality PHD filter (CPHD) [17], labelled multi-Bernoulli random finite sets (LMB RFSs) [18], Generalized LMB RFSs (GLMB RFSs) [19] and the belief theory based models [20,21,22], JPDA is still an appealing paradigm of the Bayesian data association. Many modified forms of JPDA have been developed to improving the computation complexity or performance of the JPDA equations. To solve the multiple targets tracking problem, reference [23] proposed an intuitionistic fuzzy based JPDA method. Based on the intuitionistic fuzzy point operator, a novel clustering approach of intuitionistic fuzzy is developed to obtain the intuitionistic fuzzy membership degree. Available information of measurements can be extracted by using this approach. However, the computation complexity analysis of the proposed method just compares the running time of each method. Reference [24] proposed a novel joint multi-target tracking method over a sensor network. Local joint probabilistic data association is performed by each sensor using only its own measurements. However, the calculation equations of this method are complicated and difficult to implement.

Another option to improve the JPDA method is to use the artificial intelligence method. Reference [25] proposed a modified JPDA method based on a soft and evolutionary computation method for solving the multiple targets tracking problem. The association matrix of JPDA is determined by using fuzzy evolutionary computing methods. However, the insertion of evolutionary method increases the computational complexity. Reference [26] proposed a cheap joint probabilistic data association (CJPDA) to solve multiple targets tracking problem. Furthermore, an adaptive neuro-fuzzy inference system filter is presented to finish the state update operation. However, the CJPDA method owns poor performance in the environment with dense clutters. In addition, the data association mission of multiple targets can also be considered as a feature classification problem of a candidate measurement set. Reinforcement learning (RL) is an efficient method for solving classification problems [27]. It is a trial and error procedure that an agent interacts with the environment to obtain the optimal policy to maximize a long-term reward [28,29,30]. Reference [31] introduced a deep reinforcement learning method to finish accurate target detection and association in cell tracking field. The input of a neural network is a cost matrix produced by conjointly considering various features of targets.

To overcome the aforementioned drawbacks of the classical JPDA method, this paper leverages the emerging reinforcement learning technique to handle measurement clutters, yielding a novel RL-JPDA method for the multiple targets tracking data association problem. More specifically, the proposed method uses the essential characteristics of RL to obtain available information of measurements. The distribution of measurements is defined as states of agent in RL and the agent will choose an action according to the state-action map to acquire the estimated results, which are regarded as a feedback to update its data of state-action map. Meanwhile, considering that the motion characteristics of the targets should be utilized, a corresponding metric is developed to ensure the accuracy of the association results. In addition, the learning process of each target data is independent, which means that same distribution of different targets may have different results. This approach can generate more efficient results for each target. Consequently, the main contributions of this paper include:

- The RL is embedded into the traditional JPDA method to obtain the relationship between the measurement distribution and its associated probability at the presence of dense measurement clutters;
- The motion characteristics of the targets is considered to improve the accuracy of data association.

The structure of this paper is organized as follows. The problem formulation is described in Section 2. Section 3 explains detailed implementation of the proposed RL-JPDA method. In Section 4, the experiments are introduced and comparative results with other JPDA variants are presents. Finally, Section 5 summarizes the conclusions.

## 2. Problem Formulation

### 2.1. The Target Model

It is assumed that there are t=1,2,…,T targets observed by the sensor, and the dynamics and measurement model of target are defined as follows:

(1) $$X^{t}(k)=F^{t}(k)X^{t}(k-1)+w^{t}(k)$$

(2) $$Z^{t}(k)=H^{t}(k)X^{t}(k)+v^{t}(k)$$

where $X^{t}(k)$ represents the state vector of target t at scan k, and $Z^{t}(k)$ represents the measurement vector. $F^{t}(k)$ denotes the state transition matrix, $H^{t}(k)$ denotes the measurement transition matrix. The process noise $w^{t}(k)$ is Gaussian white noise with the covariance $Q^{t}(k)$ and zero mean. The measurement noise $v^{t}(k)$ is zero mean Gaussian noise with known covariance $R^{t}(k)$.

In a clutter-free environment, the state vector of each target t is predicted and updated based on correct measurements as follows [15]:

(3) $${\hat{X}}^{t}(k|k-1)=F^{t}(k)X^{t}(k-1|k-1)$$

(4) $${\hat{P}}^{t}(k|k-1)=F{\left(k\right)}^{t}P^{t}(k-1|k-1){\left(F^{t}(k)\right)}^{T}+Q^{t}(k)$$

(5) $${\tilde{Z}}^{t}(k)=Z^{t}(k)-H^{t}(k){\hat{X}}^{t}(k|k-1)$$

(6) $$S^{t}(k)=H^{t}(k){\hat{P}}^{t}(k|k-1)H^{t}(k)+R^{t}(k)$$

(7) $$K^{t}(k)={\hat{P}}^{t}(k|k-1){\left(H^{t}(k)\right)}^{T}{\left(S^{t}(k)\right)}^{-1}$$

(8) $${\hat{X}}^{t}(k|k)={\hat{X}}^{t}(k|k-1)+K^{t}(k){\tilde{Z}}^{t}(k)$$

(9) $${\hat{P}}^{t}(k|k)=[I-K^{t}(k)H^{t}(k)]{\hat{P}}^{t}(k|k-1)$$

where ${\hat{X}}^{t}(k|k-1)$ represents the predicted state vector of the *t*^th^ target at scan *k*, and ${\hat{P}}^{t}(k|k-1)$ denotes the predicted value of state covariance. ${\tilde{Z}}^{t}(k)$ is an innovation, $S^{t}(k)$ is the innovation covariance, $K^{t}(k)$ is the Kalman filter gain, ${\hat{X}}^{t}(k|k)$ is the estimated value of state at scan *k*, ${\hat{P}}^{t}(k|k)$ is the estimated value of state covariance.

### 2.2. Joint Probabilistic Data Association Method

The JPDA method is briefly revisited here. It is assumed that all the measurements observed by one sensor at scan *k* are Z(k). To obtain the candidate measurements, the gate centered around the predicted measurement is used to complete measurement selection:

(10) $${\left[Z(k)-{\hat{Z}}^{t}(k|k-1)\right]}^{T}S^{t}{\left(k\right)}^{-1}\left[Z(k)-{\hat{Z}}^{t}(k|k-1)\right]<\zeta$$

where ${\hat{Z}}^{t}(k|k-1)$ is the predicted measurement of the *t*^th^ target. The value of parameter ζ is the limit of the gate. Qualified measurements are defined as candidate measurements $Z_{j}^{t}(k),j=1,2,\ldots ,N_{C}^{t}$. $N_{C}^{t}$ is the maximum number of the candidate measurement value.

Due to the existence of clutters, the candidate measurements contain true measurements with more false measurements. A validation matrix is defined to describe the relationship between each target and each measurement as follows:

(11) $$\Omega =[w_{j,t}],j=1,2,\ldots ,N_{C}^{t};t=0,1,\ldots ,T$$

where

(12) $$w_{j,t}=\left\{\begin{array}{l} 1,\mathrm{if}\;j\mathrm{th}\;\mathrm{measurement}\;\mathrm{lies}\;\mathrm{in}\;\mathrm{gate}\;\mathrm{of}\;\mathrm{target}\;t\\ 0,\mathrm{otherwise} \end{array}\right.$$

The parameter t=0 means “no target”.

The joint event matrix $w_{j}^{t}(\theta (k))$ is a presentation that whether joint event θ(k) contains the association of target *t* and measurement *j.* The joint event matrix is generated according to (11) and two basic hypotheses:

- Each measurement is assigned to one target uniquely.
- Each target has one measurement at most.

The posterior probabilities of the joint events are computed to explain that candidate measurements may be originated from more than one target. The posterior probabilities $P\left(\theta (k)/Z^{k}\right)$ are defined as follows:

(13) $$P\left(\theta (k)/Z^{k}\right)=\frac{1}{\varsigma }\frac{\phi !}{V^{\phi }}\prod_{j=1}^{N_{C}^{t}}{\left\{N_{tj}\left[Z_{j}(k)\right]\right\}}^{\tau_{j}}\prod_{t=1}^{T}{\left(P_{D}^{t}\right)}^{\delta_{t}}{\left(1-P_{D}^{t}\right)}^{1-\delta_{t}}$$

where $Z^{k}={\left\{Z_{l}\right\}}_{l=1}^{k}$ is the cumulative list of candidate measurements up to scan *k*, ς is a normalized constant, ϕ is the number of clutter measurements, V is the volume of the tracking gate, $N_{tj}\left[Z_{j}(k)\right]$ denotes the probability density function of the predicted measurements from target t, $\delta_{i}$ is defined as a target indicator that whether there is a measurement associated with a target $t(\delta_{t}=1)$, or not $\left(\delta_{t}=0\right)$, $\tau_{j}$ is defined as the number of targets associated with measurement j, $P_{D}$ is defined as the detection probability of the *t*^th^ target.

Therefore, the probability that measurement *j* is associated with the *t*^th^ target is shown as follows:

(14) $$\beta_{j}^{t}(k)=\sum_{\theta (k)}P\left(\theta (k)/Z^{k}\right)w_{j}^{t}(\theta (k))$$

The estimated values of the target state and state covariance are:

(15) $${\hat{X}}_{j}^{t}(k|k)={\hat{X}}_{}^{t}(k|k-1)+K^{t}(k)\left[Z_{j}^{t}(k)-{\hat{Z}}_{}^{t}(k|k-1)\right]\;$$

(16) $${\hat{X}}^{t}(k|k)=\sum_{j=0}^{N_{C}^{t}}\beta_{j}^{t}(k){\hat{X}}_{j}^{t}(k|k)$$

(17) $${Χ}_{0}^{t}(k|k)={\hat{X}}^{t}(k|k){\left({\hat{X}}_{0}^{t}(k|k)\right)}^{T}$$

(18) $${\hat{P}}^{t}(k|k)={\hat{P}}^{t}(k|k-1)-\left(1-\beta_{0}^{t}(k)\right)K^{t}(k)S^{t}(k){\left(K^{t}(k)\right)}^{T}+\sum_{j=0}^{N_{C}^{t}}\beta_{j}^{t}(k)\left({\hat{X}}_{j}^{t}(k|k){\left({\hat{X}}_{j}^{t}(k|k)\right)}^{T}-X_{0}^{t}(k|k)\right)$$

The posterior probabilities $P\left(\theta (k)/Z^{k}\right)$ need to calculate the cumulative value of all probability density functions. It is obvious that the computational cost of all joint events will increase exponentially with the increase of measurements. Meanwhile, $V^{\phi }$ will be nearly zero when the number of clutter measurements increases significantly, and the dimension explosion problem will occur.

### 2.3. Reinforcement Learning

RL has made a number of significant breakthroughs over the passage of time. Two kinds of method for solving RL problems have been divided as follows: on-policy and off-policy methods [32]. On-policy methods make decisions and evaluate the policy. However, the policy evaluated may be irrelevant to the policy used to generate data. The data used can be generated offline by applying the policy to the system, but the learning process for the policy is online. Thus, in off-policy methods, these two functions are separated. The off-policy methods reuse the experience acquired from performing policy to update value functions, which means high efficiency and speediness. Q-learning is a typical off-policy RL method, which is used widely due to its simplicity [33]. In Q-learning, action is performed with the highest expected Q-values at each state, then the agent can receive feedback from the environment, and the policy will be improved. The Q-value is updated based on the reward as follows:

(19) $$Q(s_{t},a_{t})\leftarrow Q(s_{t},a_{t})+\lambda [r_{t+1}+\gamma \underset{a}{\max}Q(s_{t+1},a)-Q(s_{t},a_{t})]$$

where $a_{t}$ is the current action, $s_{t}$ is the current state, γ is a discount parameter, $s_{t+1}$ is the next state, λ is the learning rate, $r_{t+1}$ is the RL reward acquired from the performing of $a_{t}$ at $s_{t}$, $Q(s_{t+1},a)$ is the estimated Q-value when the action a is performed at state $s_{t+1}$. The pseudocode of the Q-learning method is shown in Algorithm 1:
**Algorithm 1.** The Q-learning method pseudocode.**Initialize** Set the state s and the action a **For** each state $s_{i}$ and action $a_{i}$ Set $Q(s_{i},a_{i})=0$ **End For** Randomly choose an initial state $s_{t}$ **While** the terminal condition is not reached do Choose the best action $a_{t}$ from the current state $s_{t}$ from Q-table Execute action $a_{t}$, then get the immediate reward Find out the new state $s_{t+1}$ Acquire the corresponding maximum Q-value of $s_{t+1}$ Update the Q-table by (19) Update the state $s_{t}\leftarrow s_{t+1}$ **End While**

## 3. The Proposed RL-JPDA Method

### 3.1. RL-JPDA Development and Implementation

This section mainly explains the procedure of the proposed data association method RL-JPDA, which includes three major parts. After initialing the basic RL and JPDA parameters, for each scan, the candidate measurements and their distribution are acquired in Part 1. Then we calculate the association probability according to the target motion characteristics and candidate measurement distribution in Part 2. RL is leveraged to make full use of the distribution law of candidate measurements in this step. The tracked targets are defined as the agents of RL, and eight areas have been considered as the states in the Q-table. All agents switch action adaptively according to the distribution law. If the performing of action results owns better performance, a positive reward will be given, otherwise the punishment would be completed by giving a negative reward. In Part 3, the data association process is performed, and the Q-table is update.

**Figure 1:**
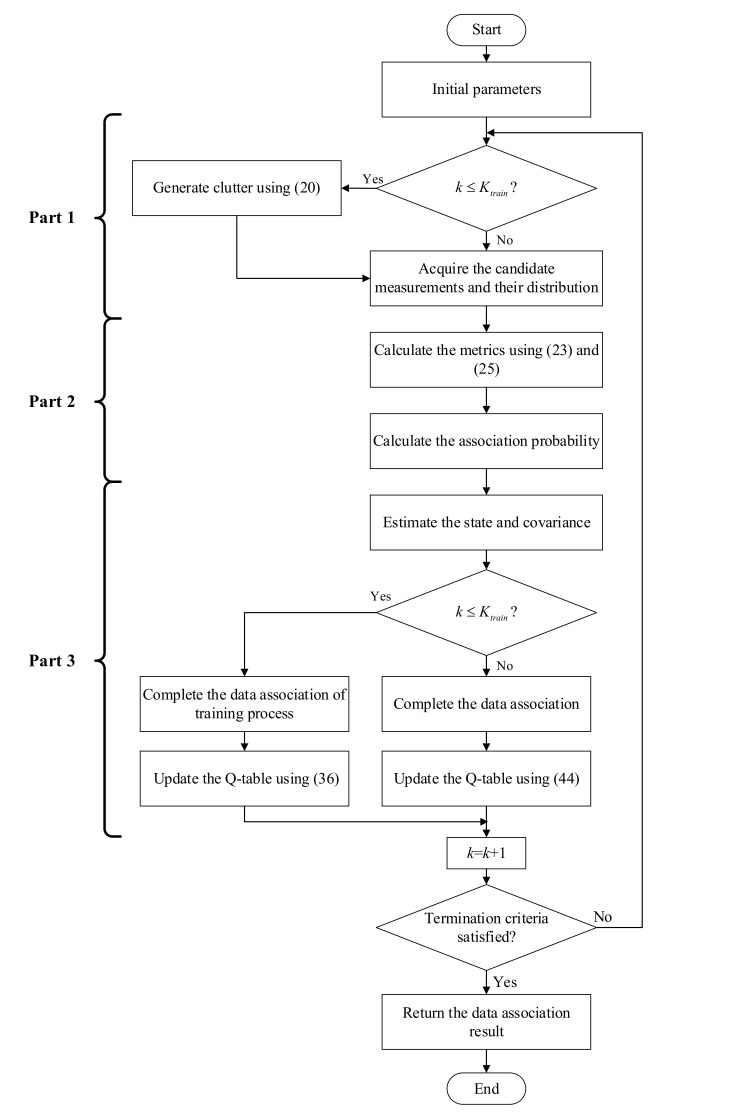
The flow chart of the reinforcement learning-joint probabilistic data association (RL-JPDA) method.

The flow chart of the RL-JPDA method is shown in Figure 1, and the pseudocode is illustrated in Algorithm 2. The detailed formulation is elaborated as follows.
**Algorithm 2.** The pseudocode for the RL-JPDA method.**Initialize** Set the basic parameters Set the state *s* ={*s*1, *s*2, *s*3, *s*4, *s*5, *s*6, *s*7, *s*8}and action *a* ={*a*1, *a*2, *a*3} Set the initial Q-table: *Q^t^*(*s*, *a*) = 0 Acquire the real measurements $Z_{}^{t}(k|k),k=1,\ldots ,K_{train}$ of the training process Set k = 1 **While**
$k<K_{\max}$
**do** **Calculating candidate measurements** **If**
$k<K_{train}$ Generate clutter $Z_{\mathrm{training}}^{}(k)$ by (20) **End If** Acquire the candidate measurements $Z_{j}^{t}(k)$ Acquire the distribution of all candidate measurements **Calculating association probability** Calculate the metric $D_{2,j}^{t}\left(k\right)$ by the (25) ** For** each candidate measurement  Choose the best *a* for the current *s* from Q-table  **Switch** action   Case 1: increase    Set the RL parameter *w* a big value   Case 2: decrease    Set the RL parameter *w* a small value   Case 3: maintain    Set the RL parameter *w* = 1 **  End Switch** **End For** Calculate the metric $D_{1,j}^{t}\left(k\right)$ by (23) Calculate the association probability by (29) **Data association and Q-table update** Estimate the state $X^{t}(k|k)$ and covariance $P^{t}(k|k)$ by (30) and (9) **If**
$k<K_{train}$ Estimate the state $X_{train}^{t}(k\left|k\right.)$ by (31) Complete the data association of training process with $X_{train}^{t}(k\left|k\right.)$  Calculate the cost value $f_{train}^{t}(k)$ by (32)  Calculate the reward $r_{train}^{t}(k)$ by (33)  Update the Q-table by (34) ** Else**  Complete the data association with $X^{t}(k|k)$  Calculate the cost value $f_{}^{t}(k)$ by (39)  Calculate the reward $r_{}^{t}(k)$ by (40)  Update the Q-table by (41) ** End If** k=k+1 **End While** **Return results** **Terminate**

#### 3.1.1. Calculating Candidate Measurements

What this paper mainly focuses on is the situation that the initial segment of multiple target tracking is clutter free, then the subsequent measurements will be mixed with clutters [34]. Thus, the targets data association of initial segment is regarded as the RL training process. During the training process, the state-action map of RL will be established preliminarily. The proposed method reconstructs the compute mode of joint association probabilities in JPDA by the state-action map of RL to acquire the available information of measurements. When the target enters the clutter region, the agent of RL will choose an action to acquire the data association estimated results according to the state-action map, and the estimated results are used to update the state-action map to ensure the accuracy of the subsequent association process. This application situation is mainly aimed at a scenario where there is no off-line training time, and the training process can also be performed offline to obtain the state-action map if the condition permits. As a result, the proposed method can be applied to the whole tracking process with dense clutters accordingly.

In the training process, the clutter $Z_{\mathrm{training}}^{}(k)$ at k scan are generated according to the measurement $Z_{}^{t}(k|k),k=1,\ldots ,K_{train}$:

(20) $$\left\{\begin{array}{l} Z_{flase,i}^{t}(k)=Z_{}^{t}(k|k)+l-2l\cdot rand_{0,1}\\ Z_{training}^{}(k)=\left\{Z_{flase,i}^{t}(k)|i\in [1,N_{f}],t\in [1,T]\right\} \end{array}\right.$$

where $i=1,2,\ldots ,N_{f}$ represents the number of clutter, l represents the gate side length, and $rand_{0,1}$ is a random parameter limited in [0,1]. $K_{train}$ is defined as the upper bound of time epochs of the training process.

Therefore, the measurements at k scan can be defined as follows:

(21) $$Z(k)=\left\{\begin{array}{l} Z_{training}^{}(k),if\;k\leq K_{train}\\ Z(k)\;,otherwise \end{array}\right.$$

**Figure 2:**
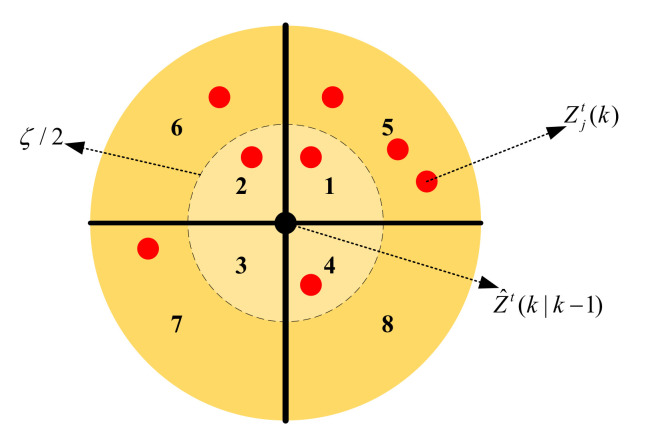
The tracking gate partition.

The candidate measurements $Z_{j}^{t}(k),j=1,2,\ldots ,N_{C}^{t}$ can be acquired by using (10). As shown in Figure 2, the tracking gate is established as a circular area, with the predicted value as the origin, ζ value given in (10) as the radius and is divided into four portions. An extra separation boundary of ζ/2 is introduced, and thus generates eight subregions of the tracking gate, which represent eight RL state values.

Therefore, the distribution of each candidate measurement can be acquired. Furthermore, the measurement distribution matrix is defined as follows:

(22) $$M_{d}^{t}=[M_{j}^{t}],j=1,2,\ldots ,N_{C}^{t},t=1,2,\ldots ,T$$

where $M_{j}^{t}$ represents the distribution of the *j*^th^ measurement.

**Figure 3:**
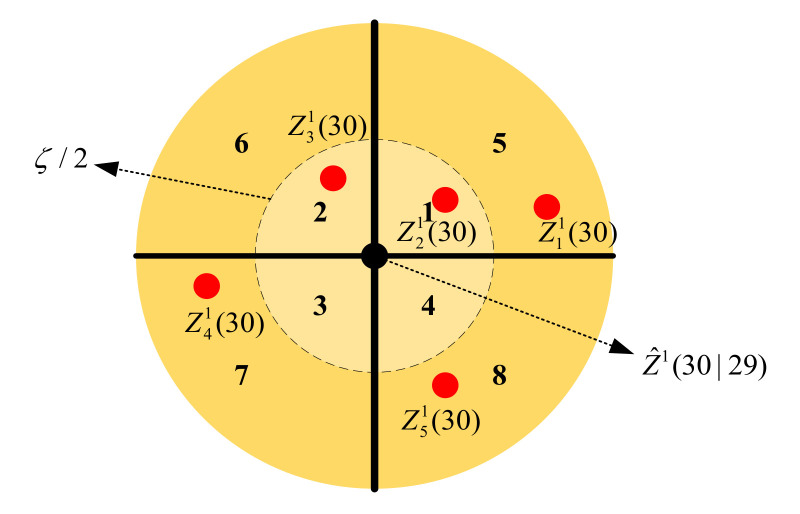
Example of the first target with five candidate measurements.

For example, the first target (t=1) has five candidate measurements ($N_{C}^{1}=5$) at the time epoch of k=30, and the distribution of each candidate measurement is shown in Figure 3. From Figure 3, the measurement $z_{1}^{1}(30)$ falls in the fifth region, i.e., ($M_{1}^{1}=5$) and $z_{2}^{1}(30)$ falls in the first region ($M_{2}^{1}=1$). The measurement $z_{3}^{1}(30)$ falls in the second region ($M_{3}^{1}=2$) and $z_{4}^{1}(30)$ falls in the seventh region ($M_{4}^{1}=7$). The measurement $z_{5}^{1}(30)$ falls in the eighth region ($M_{5}^{1}=8$). The measurement distribution matrix for Figure 3 is given as $M_{d}^{1}=[5\;1\;2\;7\;8]$.

#### 3.1.2. Calculating Association Probability

The association probability between the *j*^th^ measurement and the *t*^th^ target is calculated according to two metrics $D_{1,j}^{t}\left(k\right)$ and $D_{2,j}^{t}\left(k\right)$ defined in this work. The Mahalanobis distance between the predicted measurement and each candidate measurement is considered as the basic cost value, which is calculated as follows:

(23) $$D_{1,j}^{t}\left(k\right)=w{\left[{\hat{Z}}_{}^{t}(k\left|k-1\right.)-Z_{j}^{t}(k)\right]}^{T}S^{t}{\left(k\right)}^{-1}\left[{\hat{Z}}_{}^{t}(k\left|k-1\right.)-Z_{j}^{t}(k)\right]$$

where w is the RL parameter.

**Figure 4:**
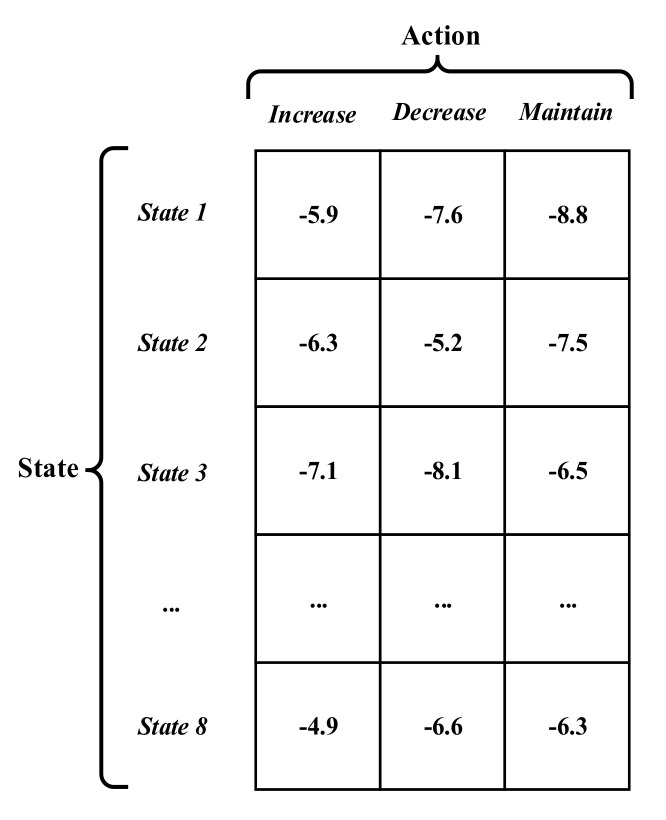
The form of Q-table.

Each basic cost value is affected by its distribution $M_{d}^{t}$ of measurement as well as the method of Q-learning. Figure 4 illustrates the form of the Q-table. The Q-table is designed as an 8 × 3 matrix. The rows of the Q-table represent the state and the columns represent the action. For each state, three actions are proposed to control the RL parameter w as follows.

- Increase action: It takes place as a result of agent lack of self-confidence. This action commonly happens when the agent finds itself fail in some scan. This failure is defined that the agent obtains a cost value defined in (23) at scan *k* that is worse than its value at scan *k* − 1. This decreases its own confidence and hence increases its RL parameter.
- Decrease action: Agent’s success may motivate such action and it reflects right decision taken by the agent, and hence, it should increase its confidence.
- Maintain action: The current RL parameter maintains the present status as there is no motivation for neither increasing nor decreasing it.

The above-mentioned three actions will directly affect the metric $D_{1,j}^{t}\left(k\right)$ as follows:

(24) $$w=\left\{\begin{array}{l} 1+\Delta \;\left(increaseaction\right)\\ 1-\Delta \;\left(decreaseaction\right)\\ 1\;\left(maintainaction\right) \end{array}\right.$$

where Δ is a change factor.

The metric $D_{2,j}^{t}\left(k\right)$ is to calculate the degree of matching between each candidate measurement and kinetic characteristic of target in the form of Mahalanobis distance $D_{2,j}^{t}\left(k\right)$:

(25) $$D_{2,j}^{t}\left(k\right)={\left[{\hat{Z}}_{k-\nu \to k}^{t}(k\left|k-\nu \right.)-Z_{j}^{t}(k)\right]}^{T}S^{t}{\left(k\right)}^{-1}\left[{\hat{Z}}_{k-\nu \to k}^{t}(k\left|k-\nu \right.)-Z_{j}^{t}(k)\right]$$

where ${\hat{Z}}_{k-\nu \to k}^{t}(k\left|k-\nu \right.)$ is the predicted measurement at the *k*^th^ scan calculated by the state vector ${\hat{X}}_{k-\nu \to k}^{t}(k\left|k-\nu \right.)$ of the *t*^th^ target at the (k−ν)^th^ scan as follows:

(26) $${\hat{X}}_{k-\nu \to k}^{t}(k\left|k-\nu \right.)=F^{t}(k)\left[F^{t}(k-1)\cdots \right.\left.\left(F^{t}(k-\nu +1)X_{}^{t}(k-\nu \left|k-\nu \right.)\right)\right]$$

(27) $${\hat{Z}}_{k-\nu \to k}^{t}(k\left|k-\nu \right.)=H^{t}(k){\hat{X}}_{k-\nu \to k}^{t}(k\left|k-\nu \right.)$$

where ν is the procedure parameter.

**Figure 5:**
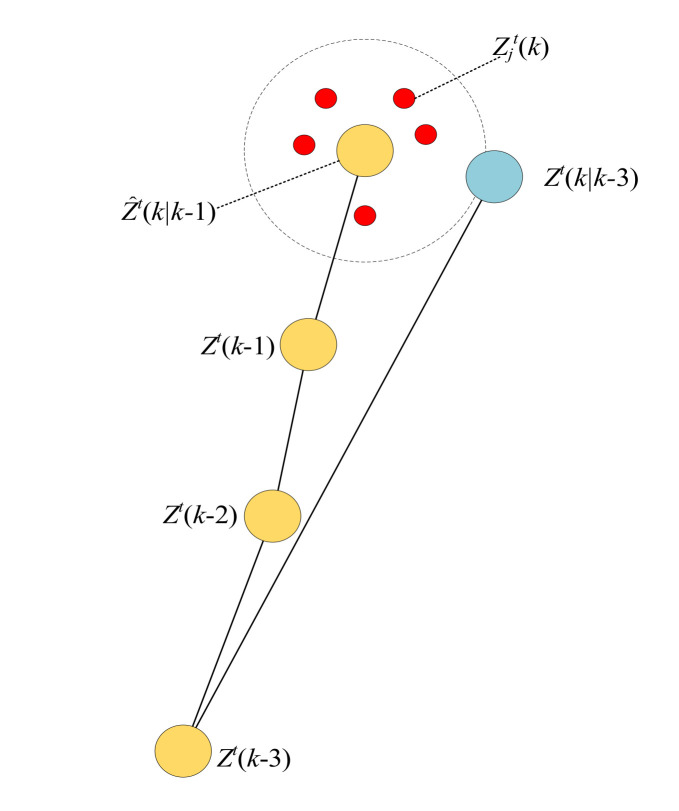
The computational process of the metric $D_{2,j}^{t}\left(k\right)$.

Figure 5 shows the computational process of the metric $D_{2,j}^{t}\left(k\right)$ when ν=3. The predicted measurement ${\hat{Z}}_{k-3\to k}^{t}(k\left|k-3\right.)$ can be calculated by (26) and (27). Then the metric $D_{2,j}^{t}\left(k\right)$ can be acquired by calculating the Euclidean distance between ${\hat{Z}}_{k-3\to k}^{t}(k\left|k-3\right.)$ and $Z_{j}^{t}(k)$. Metric $D_{2,j}^{t}\left(k\right)$ will be smaller if the measurement $Z_{j}^{t}(k)$ is more in line with the motion characteristics of the target. Otherwise, $D_{2,j}^{t}\left(k\right)$ would be amplified. Therefore, the association probability of each candidate measurement at k scan is calculated as follows:

(28) $$\beta_{j}^{t}\left(k\right)=\frac{1}{\left(D_{1,j}^{t}\left(k\right)+D_{2,j}^{t}\left(k\right)\right)}$$

(29) $$\beta_{j}^{t}\left(k\right)=\beta_{j}^{t}\left(k\right)/\sum_{j=1}^{N_{C}^{t}}\beta_{j}^{t}\left(k\right)$$

In addition, the association probability has been normalized by (29).

#### 3.1.3. Data Association and Q-table Update

According to (7) and (9), the Kalman filter is used to estimate the next state of the target as follows:

(30) $$X^{t}(k|k)=\sum_{j=1}^{m_{k}}\beta_{j}^{t}(k)({\hat{X}}_{}^{t}(k|k-1)+K^{t}(k)\left(Z_{j}^{t}(k)-{\hat{Z}}_{}^{t}(k|k-1)\right)$$

When the target enters the clutter region, the estimated results are used to complete the data association and Q-table update. However, in the training process, the result of state estimation will only be used to update Q-table. The real measurement is used to estimate the next state $X_{train}^{t}(k\left|k\right.)$ and complete the data association according to the Kalman filter as follows:

(31) $$X_{train}^{t}(k\left|k\right.)={\hat{X}}_{j}^{t}(k|k-1)+K^{t}(k)\left(Z_{}^{t}(k|k)-H^{t}(k){\hat{X}}_{j}^{t}(k|k-1)\right)$$

For the training process, the Euclidean distance between $X^{t}(k|k)$ and $X_{train}^{t}(k\left|k\right.)$ is designed as the cost value $f_{train}^{t}(k)$:

(32) $$f_{train}^{t}(k)={\left[X^{t}(k\left|k\right.)-X_{train}^{t}(k\left|k\right.)\right]}^{T}S^{t}{\left(k\right)}^{-1}\left[X^{t}(k\left|k\right.)-X_{train}^{t}(k\left|k\right.)\right]$$

Furthermore, the RL reward is calculated as follows:

(33) $$r_{train}^{t}(k)=\left\{\begin{array}{l} 1\;,\mathrm{if}\;f_{train}^{t}(k)-f_{train}^{t}(k-1)\leq 0\\ -1\;,\mathrm{otherwise} \end{array}\right.$$

Then the Q-table is updated as follows:

(34) $$Q^{t}(s_{i},a_{j})=Q^{t}(s_{i},a_{j})+\lambda \left[r_{train}^{t}(k)+\right.\left.\gamma \underset{a}{\max}Q^{t}(s_{i},a_{})-Q^{t}(s_{i},a_{j})\right]$$

where i=1,2,…,8 is the number of RL states. When the target enters the clutter region, the predicted state ${\hat{X}}_{}^{t}(k|k-1)\;$and state estimation $X_{}^{t}(k|k)$ at the ${\left(k+1\right)}^{\mathrm{th}}$ scan are calculated as follows:

(35) $${\hat{X}}^{t}(k+1\left|k-1\right.)=F^{t}(k+1){\hat{X}}_{}^{t}(k|k-1)$$

(36) $${\hat{X}}^{t}(k+1\left|k\right.)=F^{t}(k+1)X_{}^{t}(k|k)$$

The predicted measurements of ${\hat{X}}_{}^{t}(k|k-1)\;$ and $X_{}^{t}(k|k)$ at the ${\left(k+1\right)}^{\mathrm{th}}$ scan are calculated as follows:

(37) $${\hat{Z}}^{t}(k+1\left|k-1\right.)=H^{t}(k+1){\hat{X}}_{}^{t}(k+1\left|k-1\right.)$$

(38) $${\hat{Z}}^{t}(k+1\left|k\right.)=H^{t}(k+1){\hat{X}}_{}^{t}(k+1\left|k\right.)$$

The Mahalanobis distance between the predicted measurements ${\hat{Z}}^{t}(k+1\left|k-1\right.)$ and ${\hat{Z}}^{t}(k+1\left|k\right.)$ is considered as the cost value $f_{}^{t}(k)$:

(39) $$f_{}^{t}(k)={\left({\hat{Z}}^{t}(k+1\left|k-1\right.)-{\hat{Z}}^{t}(k+1\left|k\right.)\right)}^{T}S_{}^{t}{\left(k+1\right)}^{-1}\left({\hat{Z}}^{t}(k+1\left|k-1\right.)-{\hat{Z}}^{t}(k+1\left|k\right.)\right)$$

where $S_{}^{t}(k+1)=H^{t}(k+1)P^{t}(k|k)H^{t}{\left(k+1\right)}^{T}$.

Furthermore, the RL reward is calculated as follows:

(40) $$r_{}^{t}(k)=\left\{\begin{array}{l} 1\;,\mathrm{if}\;f_{}^{t}(k)-f_{}^{t}(k-1)\leq 0\\ -1\;,\mathrm{otherwise} \end{array}\right.$$

Then the Q-table is updated as follows:

(41) $$Q^{t}(s_{i},a_{j})=Q^{t}(s_{i},a_{j})+\lambda \left[r_{}^{t}(k)+\gamma \underset{a}{\max}Q^{t}(s_{i},a_{})-Q^{t}(s_{i},a_{j})\right]$$

### 3.2. Computing Complexity

As shown in Figure 1, the initialization process is performed one time at the start, and the data association process is executed in each cycle. The number of targets is *T*. The number of all measurements obtained by the sensor at the *k*^th^ scan is *M*. The number of all candidate measurements at the *k*^th^ scan is $N_{C}^{t}$. For the initialization phase, the basic parameters are initialized, and the corresponding computing complexity is O(1). Then, the method starts to perform data association.

In Part 1, *M* measurements include real measurements and generated clutters. The computing complexity of generating clutters is O(M−T). Furthermore, the computing complexity of acquiring candidate measurements is O(M⋅T) of each scan. In Part 2, the metric $D_{2,j}^{t}\left(k\right)$ mainly calculates the degree of matching between each candidate measurement and kinetic characteristic of target. The computing complexity of this operation is $O(N_{C}^{t})$. The metric $D_{1,j}^{t}\left(k\right)$ needs to obtain the RL parameter and the Euclidean distance between the predicted measurement and each candidate measurement. The computing complexity of metric $D_{1,j}^{t}\left(k\right)$ is shown as follows:

(42) $$O(\sum_{t=1}^{T}N_{C}^{t})+O(N_{C}^{t})=O(\sum_{t=1}^{T}N_{C}^{t})$$

The computing complexity of calculating association probability is $O(\sum_{t=1}^{T}N_{C}^{t})$. In Part 3, for the training process, the measurements association mainly needs to acquire three parts: estimated covariance, estimated state calculated by the candidate measurements and estimated state calculated by the real measurements. So, the computing complexity of measurements association in the training process is shown as follows:

(43) $$O(T)+O(\sum_{t=1}^{T}N_{C}^{t})+O(T)=O(\sum_{t=1}^{T}N_{C}^{t})$$

When the target enters the clutter region, the measurements association needs to acquire two parts: estimated covariance and estimated state calculated by the candidate measurements. So, the computing complexity of measurements association is shown as follows:

(44) $$O(T)+O(\sum_{t=1}^{T}N_{C}^{t})=O(\sum_{t=1}^{T}N_{C}^{t})$$

The computing complexity of updating Q-table at each scan is $O(\sum_{t=1}^{T}N_{C}^{t})$.

Therefore, because *M* is greater than $N_{C}^{t}$, so the maximum computing complexity of the proposed method is O(M⋅T) in each scan.

## 4. The Experiments and Results

In this section, three experiments are designed to evaluate the effectiveness and feasibility of the RL-JPDA method. The comparative results with GNN [35], JPDA [15], EDA [21], FOMJPDA [36], IFJPDA1 and IFJPDA2 [23] methods are also given to show the superiority of the proposed method. The initial parameters are set as follows: The upper limit of training process $K_{train}$ is set as 16. The upper limit of scan $K_{\max}$ is set as 100. The change factor △ is set as 0.5. The procedure parameter ν is set as 3. The ellipsoid tracking gate size ζ is set as 9.21. Thirty Monte Carlo simulations are performed to acquire the experimental results.

### 4.1. Scenario of Two Targets with Constant Velocity

In this section, the clutter distributed in the field of view (FOV) of the sensor is modelled with the intensity uniformly for space tracking applications [37]:

(45) $$C(z)=\lambda_{z}U(z)$$

(46) $$U(z)=\left\{\begin{array}{l} 1/V,\mathrm{if}\;z\in \mathrm{FOV}\\ 0,\;\mathrm{if}\;z\notin \mathrm{FOV} \end{array}\right.$$

where $\lambda_{z}$ denotes the mean return rate of the measurement clutter, V is the volume of the tracking gate. Two cases are considered to compare the performance of the methods with different clutter rates ($\lambda_{z}=20$ and $\lambda_{z}=40$, respectively). The targets are assumed to move in straight lines with constant velocity. Measurement data are created by simulating the actual target motion in two dimensions and then adding noise to the true measurements. The targets state model is defined by (1) and (2), where the state transition matrix *F* and measurement matrix *H* are given by:

(47) $$F=\left[\begin{array}{cccc} 1 & \tau & 0 & 0\\ 0 & 1 & 0 & 0\\ 0 & 0 & 1 & \tau \\ 0 & 0 & 0 & 1 \end{array}\right]$$

(48) $$H=\left[\begin{array}{cccc} 1 & 0 & 0 & 0\\ 0 & 0 & 1 & 0 \end{array}\right]$$

where τ is the sampling interval.

The state vector $X^{t}(k)$ contains target positions and velocity

(49) $$X^{t}(k)={\left[\begin{array}{cccc} x(k) & \dot{x}(k) & y(k) & \dot{y}(k) \end{array}\right]}^{T}$$

where x(k) denotes the x-coordinate of target, y(k) denotes the y-coordinate of target, $\dot{x}(k)$ and $\dot{y}(k)$ denote the corresponding velocity of target respectively. The process noise and measurement noise are assumed to be Gaussian noise with zero mean and covariance *Q*, *R*:

(50) $$Q=\mathrm{cov}(w(k))=\left[\begin{array}{cc} \tau^{2}/2 & 0\\ \tau & 0\\ 0 & \tau^{2}/2\\ 0 & \tau \end{array}\right]q{\left[\begin{array}{cc} \tau^{2}/2 & 0\\ \tau & 0\\ 0 & \tau^{2}/2\\ 0 & \tau \end{array}\right]}^{T}$$

(51) $$R=\mathrm{cov}(v(k))=diag\left(\left[\begin{array}{cc} 100^{2}m^{2} & 100^{2}m^{2} \end{array}\right]\right)$$

where $q=diag\left(\left[\begin{array}{cc} 0.5^{2}m^{2}s^{4} & 0.5^{2}m^{2}s^{4} \end{array}\right]\right)$. The target detection probabilities are assumed to be 1.0 and the sampling interval is taken to be 1 s. The initial positions ((*x*, *y*) in meters) of the two targets are assumed to be (−30,500 m, 24,500 m) and (−25,250 m, 31,500 m), for Target 1 and 2, respectively.

**Figure 6:**
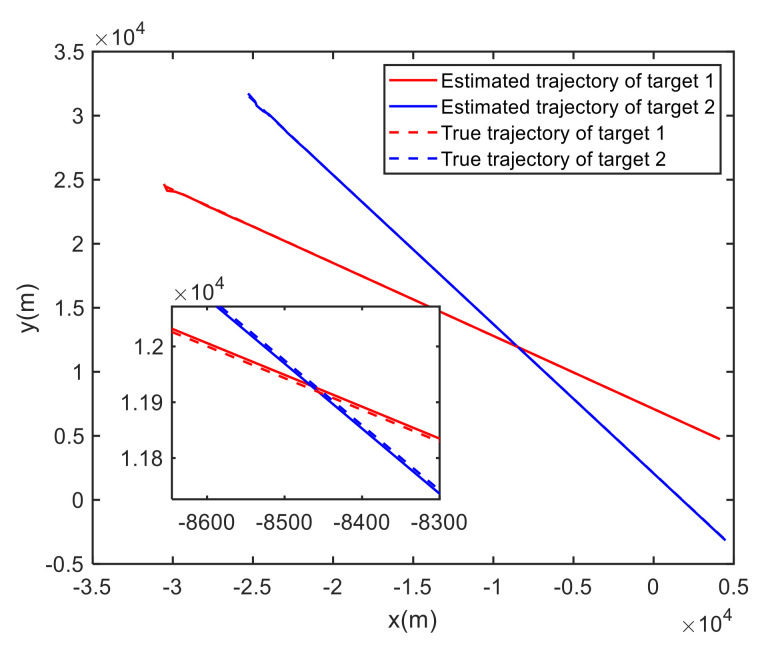
True and estimated tracks using the RL-JPDA in Case 1.

**Figure 7:**
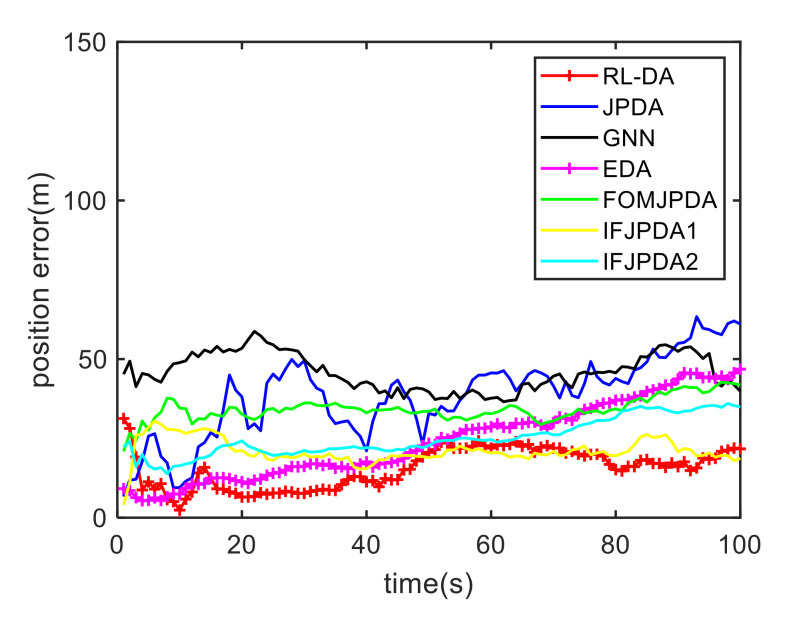
Comparison of the position errors for Target 1 in Case 1.

**Figure 8:**
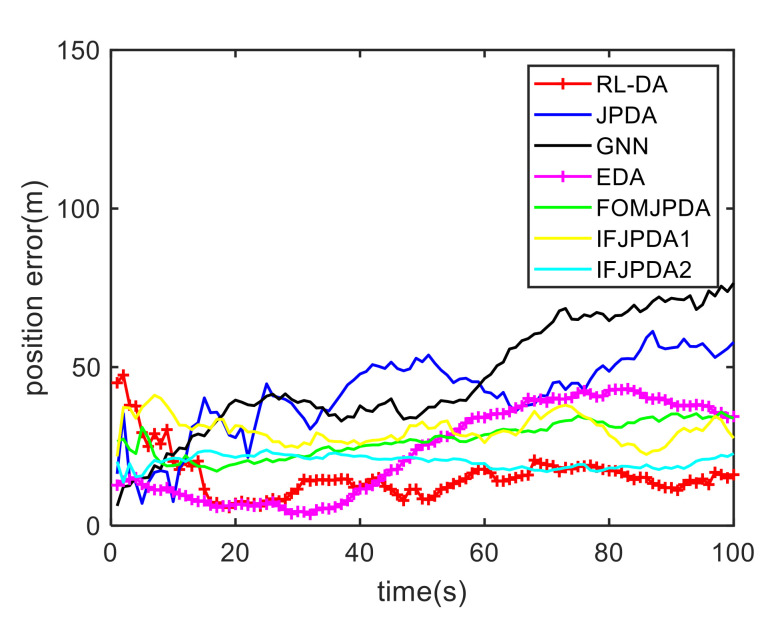
Comparison of the position errors for Target 2 in Case 1.

In Case 1, Figure 6 shows the trajectory estimation of the RL-JPDA method. It is indicated the proposed method presents better trajectory association performance. The position estimation errors of seven methods in Case 1 are illustrated in Figure 7 and Figure 8. The position error is defined as:

(52) $$e=\sqrt{e_{x}^{2}+e_{y}^{2}}=\sqrt{{\left(x_{true}-\hat{x}\right)}^{2}+{\left(y_{true}-\hat{y}\right)}^{2}}$$

where $x_{true}$ and $y_{true}$ are the real target positions, $\hat{x}$ and $\hat{y}$ are the estimated target positions. It is obvious that the proposed method performs better on the data association process than the other methods because it employs the RL and motion characteristics. The position error of the IFPDA2 method is slightly higher than the proposed method. All other methods have poor performance in Case 1.

**Figure 9:**
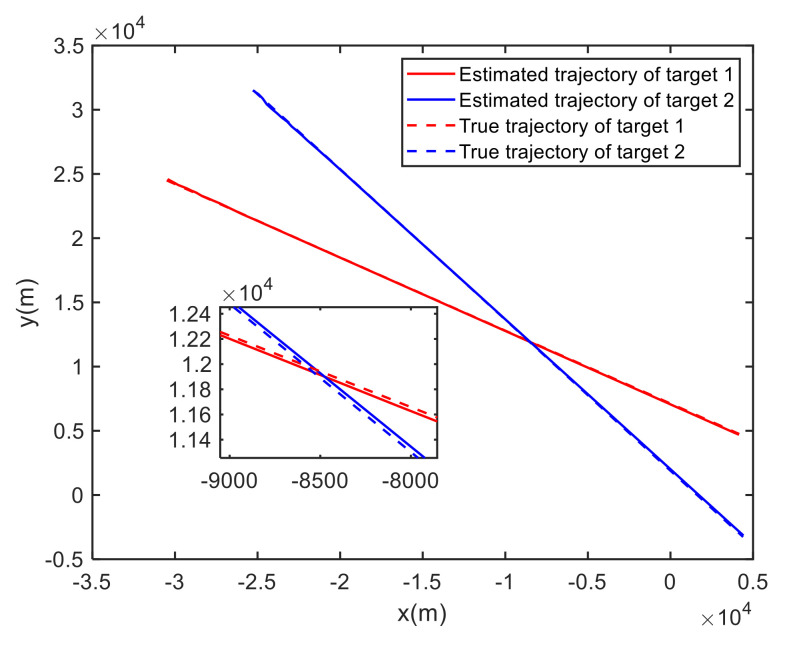
True and estimated tracks using the RL-JPDA in Case 2.

**Figure 10:**
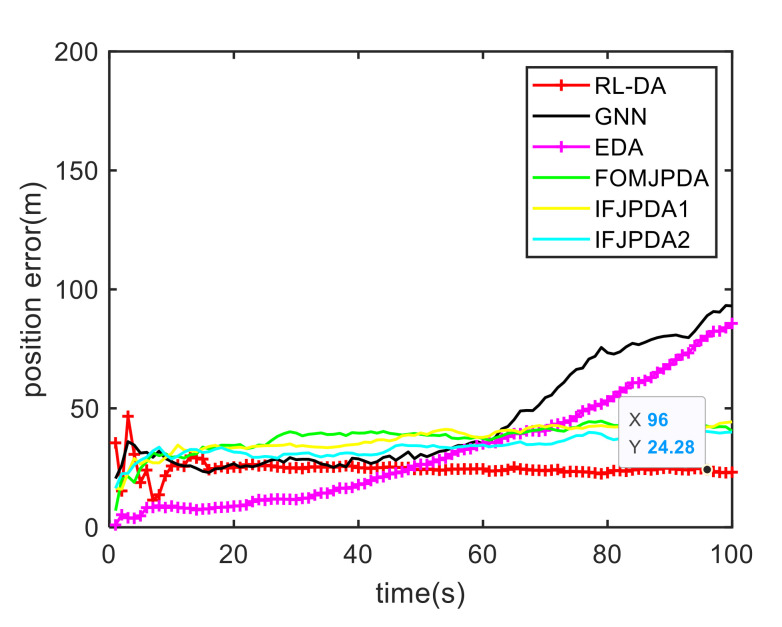
Comparison of the position errors for Target 1 in Case 2.

**Figure 11:**
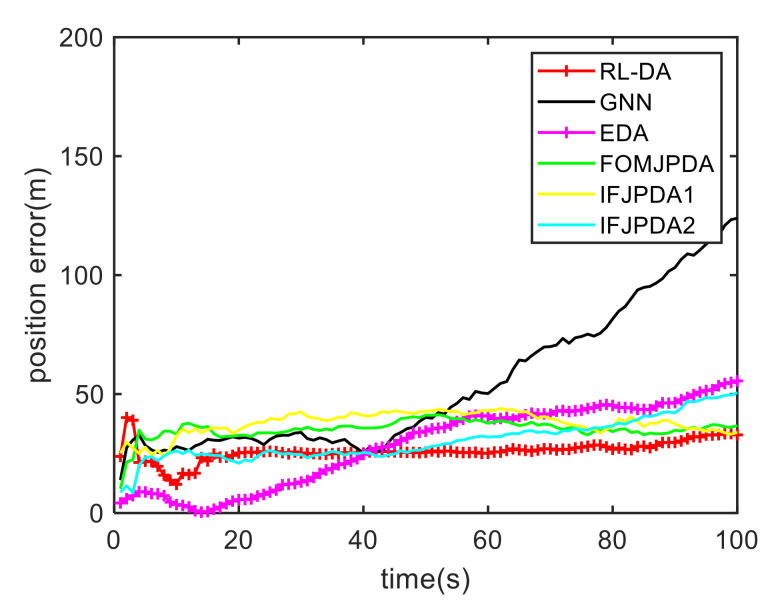
Comparison of the position errors for Target 2 in Case 2.

For the second case, we have increased the density of clutter. Because of the dimension explosion, the JPDA method cannot complete the trajectory association mission. Figure 9 shows the trajectory estimation result of the RL-JPDA method. The trajectory associated by the proposed method still presents better performance. The position errors of seven methods in Case 1 are illustrated in Figure 10 and Figure 11. The position error of other methods in Case 2 is larger than that in Case 1. This is mainly due to the association errors of targets increasing with the increment of the clutter density, which result in a performance decrease for all methods. In addition, The RL-JPDA method outperforms the GNN, JPDA, EDA, FOMJPDA, IFJPDA1 and IFJPDA2 methods with an increasing clutter density. The error results also show that the proposed method can complete the trajectory association mission accurately in dense clutter environments.

The root mean square (RMS) position errors and execution time are illustrated in Table 1 for all methods. For Case 1, the RMS errors of RL-JPDA are 16.74 m and 17.21 m, which are superior to other methods. The RMS errors of EDA are 27.15 m and 27.78 m, which are better than that of GNN, JPDA and FOMJPDA. The execution time of EDA is 0.38 s. These data indicate that EDA method has lower computational complexity and better estimated result. The RMS errors of IFJPDA2 are 25.74 m and 20.27 m, which are higher to the proposed methods slightly. The results of other methods have small error differences. For Case 2, Table 1 shows that the RMS errors of RL-JPDA are 24.90 m and 26.60 m, which are also superior to other methods significantly. The RMS results of IFJPDA1 are worse than of IFJPDA2, but the execution time of IFJPDA2 is 1.34 s. Because the degree of association is obtained by splitting the validation matrix during the computational process of IFJPDA2 method. Furthermore, this operation increases computational complexity greatly. The proposed methods do not need to perform this operation, and there is no rapid increase in the computational complexity with increasing clutter density.

### 4.2. Scenario of Three Targets with Constant Acceleration

In this section, the targets are assumed to move with a constant acceleration, and two cases with different density values of clutters are also considered to compare the performance of the methods. The state transition matrix *F* and measurement matrix *H* are given by:

(53) $$F=\left[\begin{array}{cccccc} 1 & \tau & \tau^{2}/2 & 0 & 0 & 0\\ 0 & 1 & \tau & 0 & 0 & 0\\ 0 & 0 & 1 & 0 & 0 & 0\\ 0 & 0 & 0 & 1 & \tau & \tau^{2}/2\\ 0 & 0 & 0 & 0 & 1 & \tau \\ 0 & 0 & 0 & 0 & 0 & 1 \end{array}\right]$$

(54) $$H=\left[\begin{array}{cccccc} 1 & 0 & 0 & 0 & 0 & 0\\ 0 & 0 & 0 & 1 & 0 & 0 \end{array}\right]$$

where τ is the sampling interval.

The state vector $X^{t}(k)$ contains target positions, velocity and acceleration:

(55) $$X^{t}(k)={\left[\begin{array}{cccccc} x(k) & \dot{x}(k) & \ddot{x}(k) & y(k) & \dot{y}(k) & \ddot{y}(k) \end{array}\right]}^{T}$$

where x(k) denotes the x-coordinate of target, y(k) denotes the y-coordinate of target, $\dot{x}(k)$ and $\dot{y}(k)$ denote the corresponding velocity of target, respectively, $\ddot{x}(k)$ and $\ddot{y}(k)$ denote the corresponding acceleration of target, respectively. The process noise covariance *Q* and measurement noise covariance *R* are defined as follows:

(56) $$Q=q\left[\begin{array}{cccccc} \tau^{5}/20 & \tau^{4}/8 & \tau^{3}/6 & 0 & 0 & 0\\ \tau^{4}/8 & \tau^{3}/3 & \tau^{2}/2 & 0 & 0 & 0\\ \tau^{3}/6 & \tau^{2}/2 & \tau & 0 & 0 & 0\\ 0 & 0 & 0 & \tau^{5}/20 & \tau^{4}/8 & \tau^{3}/6\\ 0 & 0 & 0 & \tau^{4}/8 & \tau^{3}/3 & \tau^{2}/2\\ 0 & 0 & 0 & \tau^{3}/6 & \tau^{2}/2 & \tau \end{array}\right]$$

(57) $$R=diag\left(\left[\begin{array}{cc} 100^{2}m^{2} & 100^{2}m^{2} \end{array}\right]\right)$$

where $q=0.1^{2}m^{2}s^{4}$.The initial positions of the three targets are assumed to be (−35,500 m, 24,500 m), (−35,550 m, 31,500 m) and (−35550 m, 0 m), for Target 1, 2 and 3, respectively.

**Figure 12:**
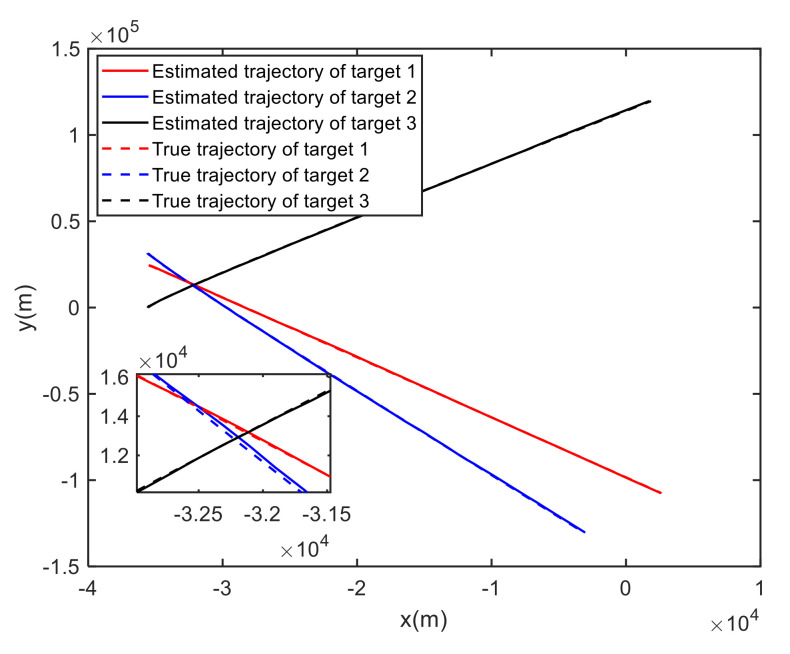
True and estimated tracks using the RL-JPDA in Case 1.

**Figure 13:**
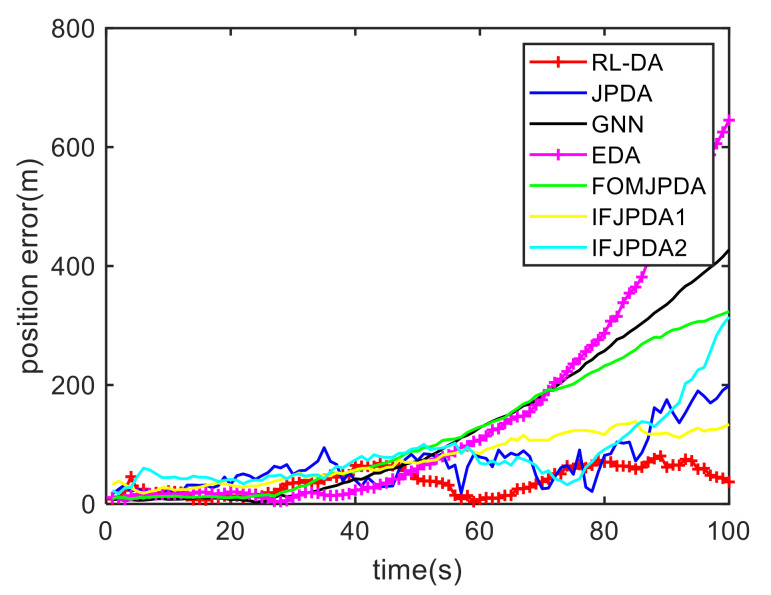
Comparison of the position errors for Target 1 in Case 1.

**Figure 14:**
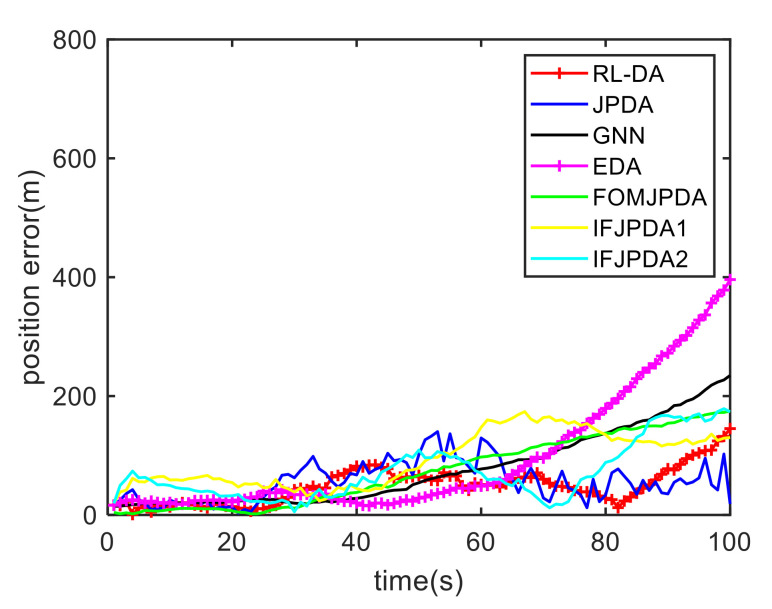
Comparison of the position errors for Target 2 in Case 1.

**Figure 15:**
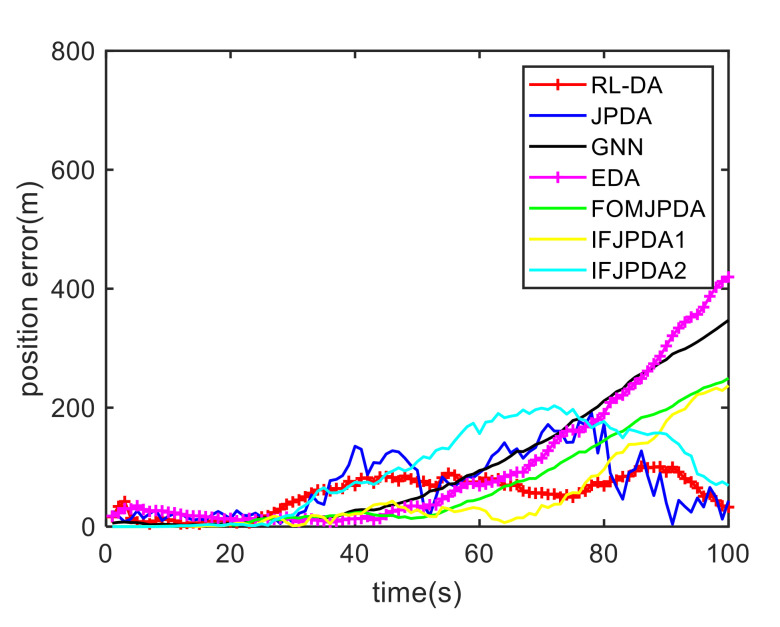
Comparison of the position errors for Target 3 in Case 1.

**Figure 16:**
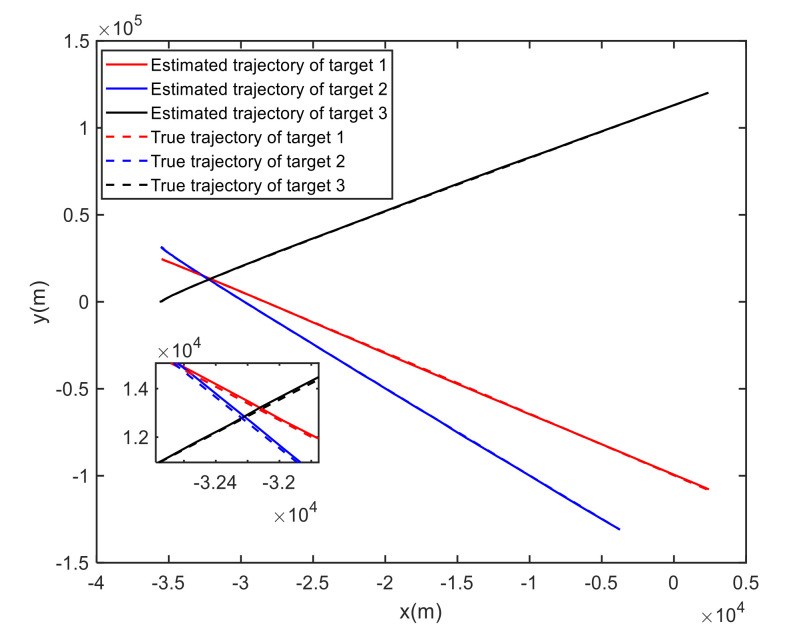
True and estimated tracks using the RL-JPDA in Case 2.

**Figure 17:**
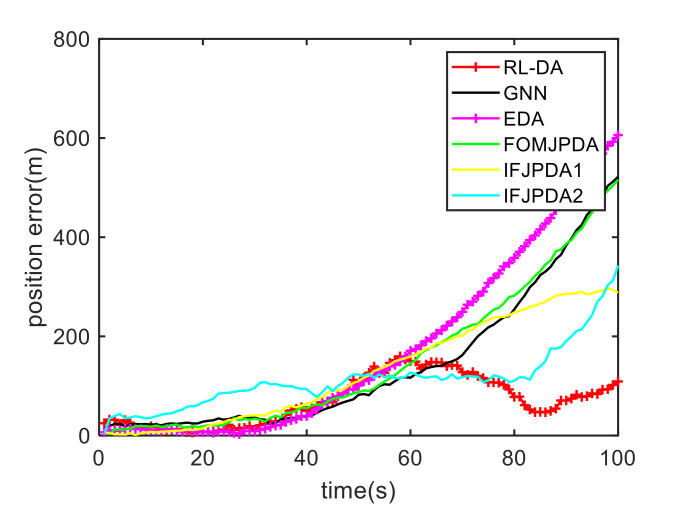
Comparison of the position errors for Target 1 in Case 2.

**Figure 18:**
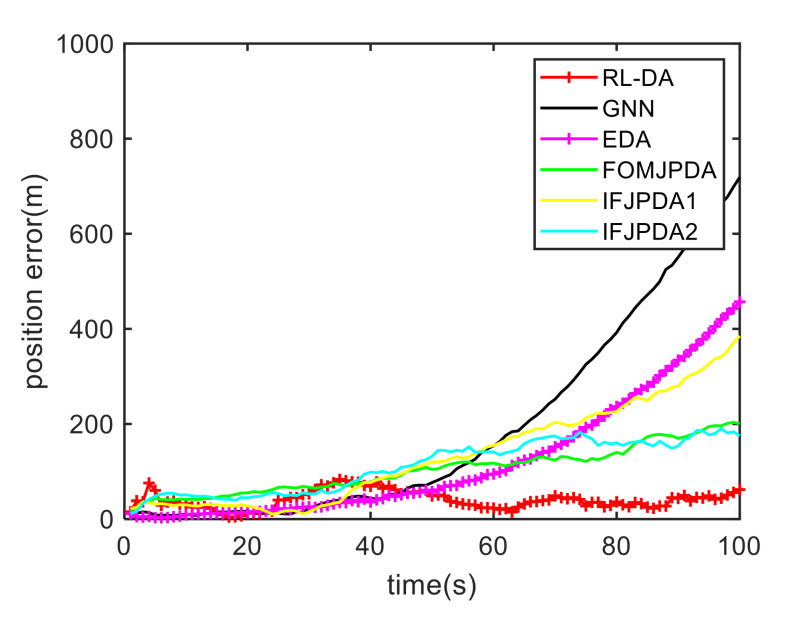
Comparison of the position errors for Target 2 in Case 2.

**Figure 19:**
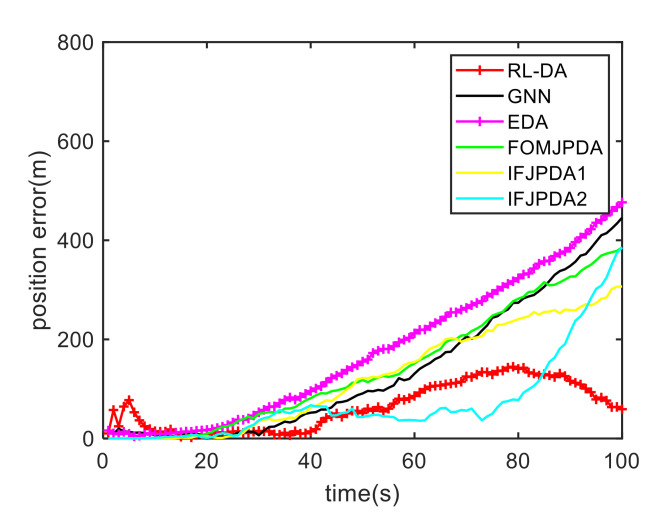
Comparison of the position errors for Target 3 in Case 2.

For Case 1, Figure 12 shows the trajectory estimation result of the RL-JPDA method. The trajectory associated by the proposed method owns significant performance. The mean position errors of the seven methods in Case 1 are illustrated in Figure 13, Figure 14 and Figure 15. It is obvious that the proposed method obtains better estimated results and achieves better performance compared to other methods. For the second case, the JPDA method still cannot complete the trajectory association mission because of the dimension explosion. Figure 16 shows the trajectory estimation result of the RL-JPDA method. The trajectory associated by RL-JPDA method owns better performance. Because the proposed method uses RL to acquire the association probability, which is different from JPDA, FOMJPDA, IFJPDA1 and IFJPDA2. Furthermore, the state estimation of targets becomes more accurate, the tracking performance is also improved. The mean position error of seven methods in Case 2 are illustrated in Figure 17, Figure 18 and Figure 19. It is obviously that the proposed method has best performance on the trajectory estimation. The other methods cannot maintain stable performance in tracking three targets.

The comparison results of the RMS errors and execution time are illustrated in Table 2. In Case 1, the RMS errors of RL-JPDA are 44.48 m, 57.21 m and 61.94 m, which are superior to those of other methods obviously. The execution time of RL-JPDA is 0.75 s, but execution time of JPDA is 4.91 s. These data indicate that the embedding of RL improves the calculation process of association probability in JPDA, and the computational complexity is greatly reduced. Meanwhile, when the target is moving with a constant acceleration, the tracking results of uniform accelerated targets are not stable by using these data association methods based on fuzzy clustering. Thus, the thirty Monte Carlo results of JPDA are better than FOMJPDA, IFJPDA1 and IFJPDA2. The EDA method has poor performance but minimum execution time. The GNN method yields maximum RMS error, which indicates that GNN method has the worst estimated result on the trajectory association of multiple targets with constant acceleration. As the clutter density increases, explosive growth in the calculation happens because the valid measurements that falls into the tracking gate increases. However, the execution time of RL-JPDA is 0.92 s, which indicates that the proposed method has lower computational complexity than other methods except for EDA.

### 4.3. Scenario of Reentry Vehicle

In this section, a reentry vehicle tracking scenario is used to verify the performance of the proposed method, and two cases with different proximity degrees of targets are also considered. Because of the strong nonlinearities exhibited by the forces of aerodynamic drag, gravity and random buffeting terms that act on the vehicle, the tracking problem of reentry vehicle is particularly stressful for data association methods. The vehicle dynamic model is [38]:

(58) $$\left\{\begin{array}{l} {\dot{x}}_{1}(k)=x_{3}(k)\\ {\dot{x}}_{2}(k)=x_{4}(k)\\ {\dot{x}}_{3}(k)=D(k)x_{3}(k)+G(k)x_{1}(k)+w_{1}(k)\\ {\dot{x}}_{4}(k)=D(k)x_{4}(k)+G(k)x_{2}(k)+w_{2}(k)\\ {\dot{x}}_{5}(k)=w_{3}(k) \end{array}\right.$$

where $x_{1}(k)$ and $x_{2}(k)$ are the position of the vehicle, $x_{3}(k)$ and $x_{4}(k)$ are the velocity of the vehicle, $x_{5}(k)$ is a parameter of its aerodynamic properties, G(k) is the gravity term, D(k) is the drag term, $w_{i}(k),i=1,2,3$ is the process noise. The force terms are given by:

(59) $$\left\{\begin{array}{l} D(k)=-\beta (k)\exp\left(\frac{\left(R_{0}-R(k)\right)}{H_{0}}\right)V(k)\\ G(k)=-\frac{Gm_{0}}{r^{3}(k)} \end{array}\right.$$

(60) $$\left\{\begin{array}{l} \beta (k)=\beta_{0}\exp\left(x_{5}(k)\right)\\ R(k)=\sqrt{x_{1}^{2}(k)+x_{2}^{2}(k)}\\ V(k)=\sqrt{x_{3}^{2}(k)+x_{4}^{2}(k)} \end{array}\right.$$

The position of the vehicle is tracked by a radar located at $\left(x_{r},y_{r}\right)$ which measures range r and bearing θ [37]:

(61) $$r(k)=\sqrt{{\left(x_{1}(k)-x_{r}\right)}^{2}+{\left(x_{2}(k)-y_{r}\right)}^{2}}+v_{1}(k)$$

(62) $$\theta (k)=\tan^{-1}\left(\frac{x_{2}(k)-y_{r}}{x_{1}(k)-x_{r}}\right)+v_{2}(k)$$

where $v_{1}(k)$ and $v_{2}(k)$ are zero-mean measurement noises.

The initial parameters of this section are set as follows: $\beta_{0}=-0.59783$, $H_{0}=13.406$, $Gm_{0}=3.9860\times 10^{5}$, $R_{0}=6374$. The initial positions of the three targets are (6500 km, 3490 km), (6500 km, 3590 km) and (6500 km, 3390 km) in Case 1. The initial positions in Case 2 are (6500 km, 3560 km), (6500 km, 3580 km) and (6500 km, 3570 km). The position of the radar is (6374 km, 0 km). Each track consists of fifty sampling time dots. The process noise covariance is $Q=diag\left(\left[\begin{array}{ccc} 2.4064\times 10^{-5}{\mathrm{km}}^{2}s^{4} & 2.4064\times 10^{-5}{\mathrm{km}}^{2}s^{4} & 0 \end{array}\right]\right)$, and measurement noise covariance is $R=diag\left(\left[\begin{array}{cc} 1^{2}m^{2} & 17^{2}{\mathrm{mrad}}^{2} \end{array}\right]\right)$. Because of the nonlinearity of the model, the unscented Kalman filter [37] is used for target state estimation.

**Figure 20:**
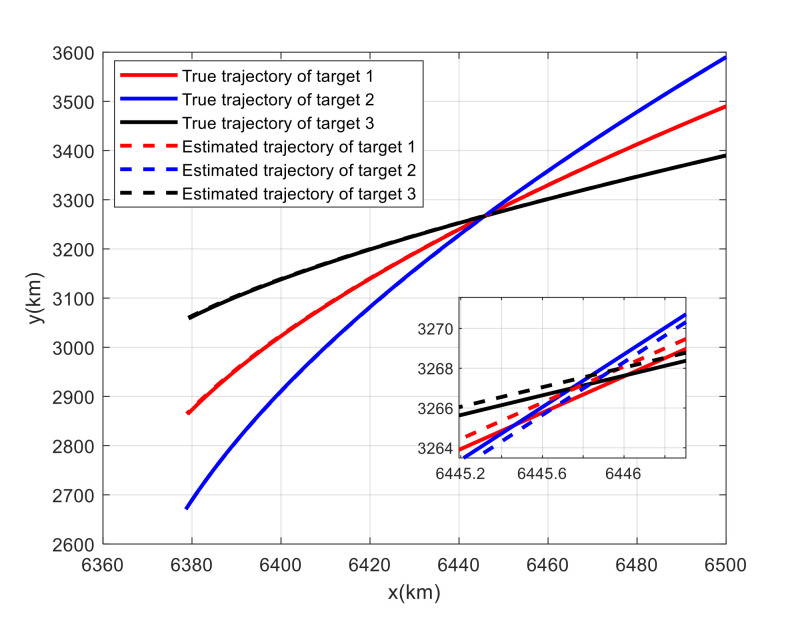
True and estimated tracks using the RL-JPDA in Case 1.

**Figure 21:**
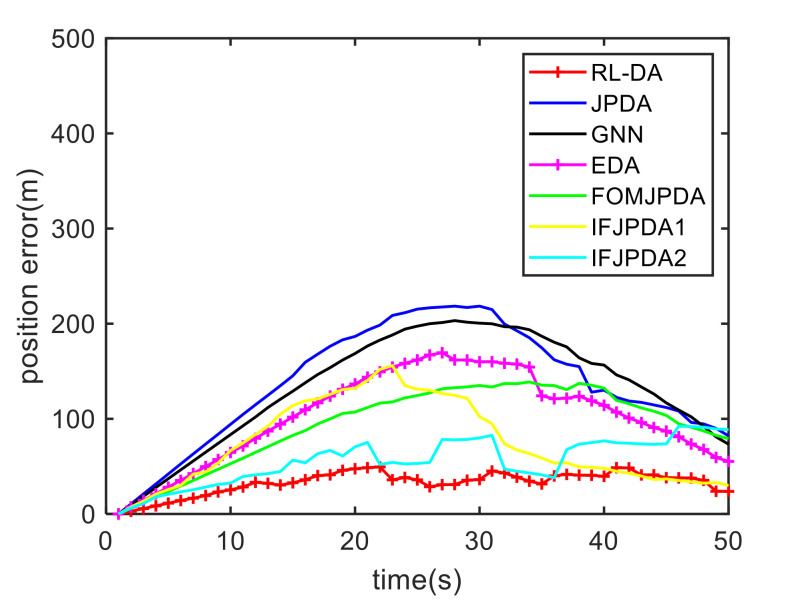
Comparison of the position errors for Target 1 in Case 1.

**Figure 22:**
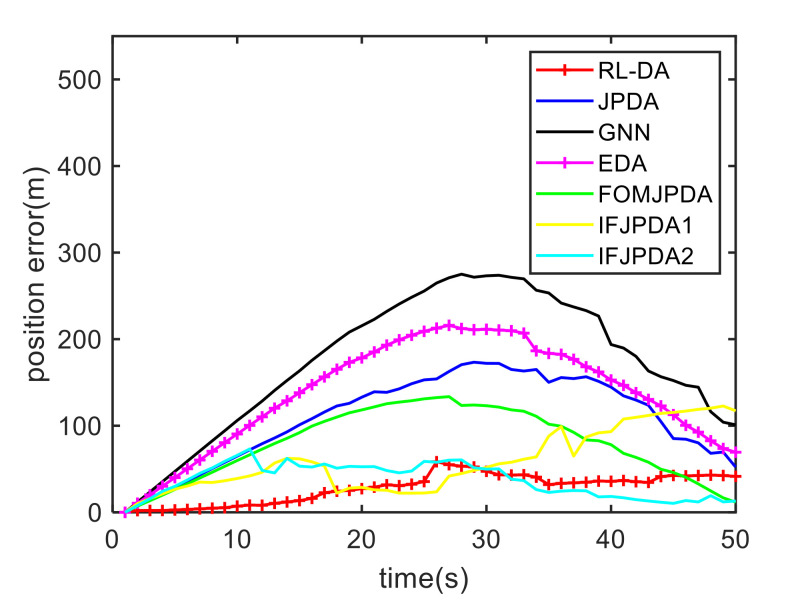
Comparison of the position errors for Target 2 in Case 1.

**Figure 23:**
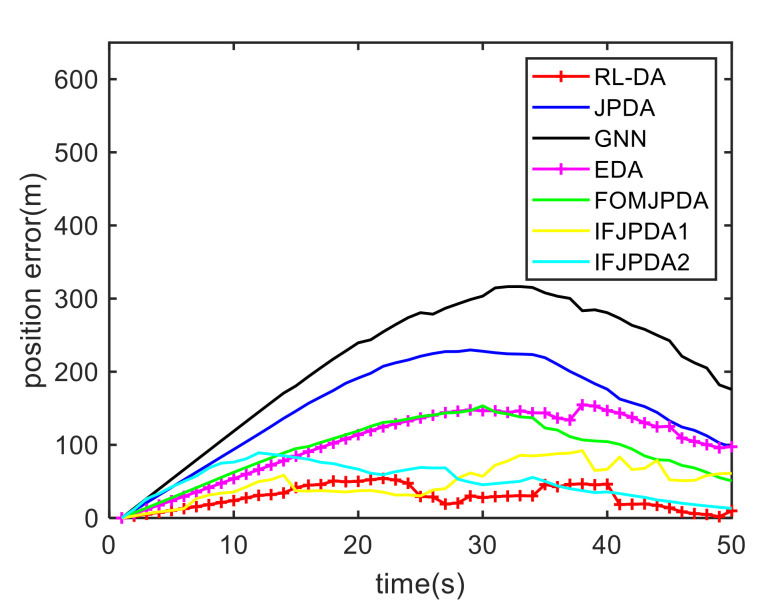
Comparison of the position errors for Target 3 in Case 1.

**Figure 24:**
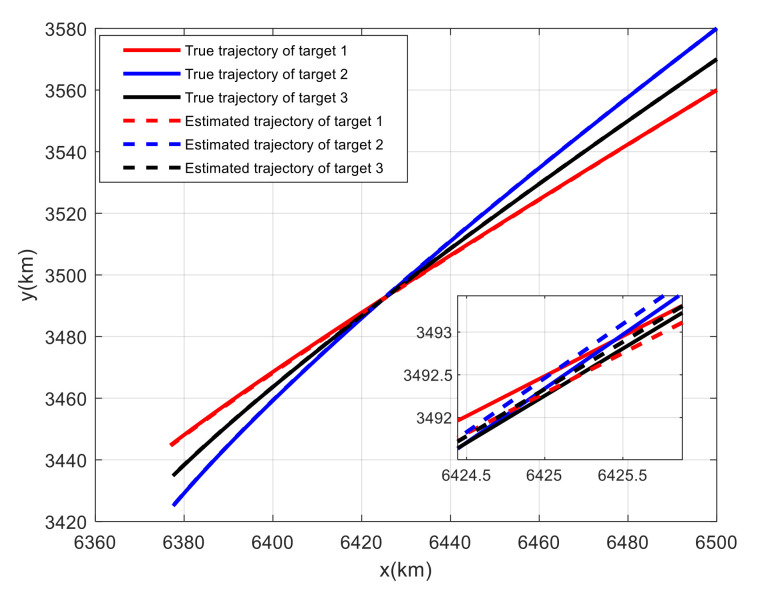
True and estimated tracks using the RL-JPDA in Case 2.

**Figure 25:**
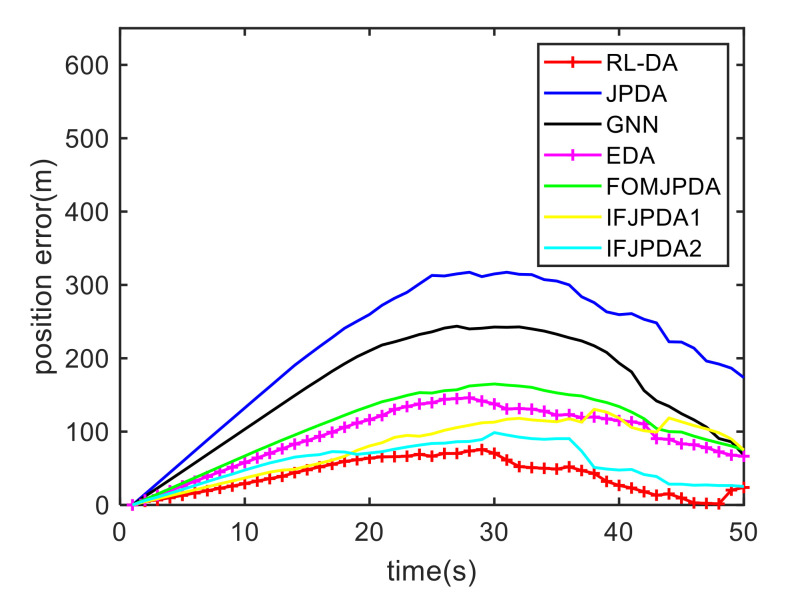
Comparison of the position errors for Target 1 in Case 2.

**Figure 26:**
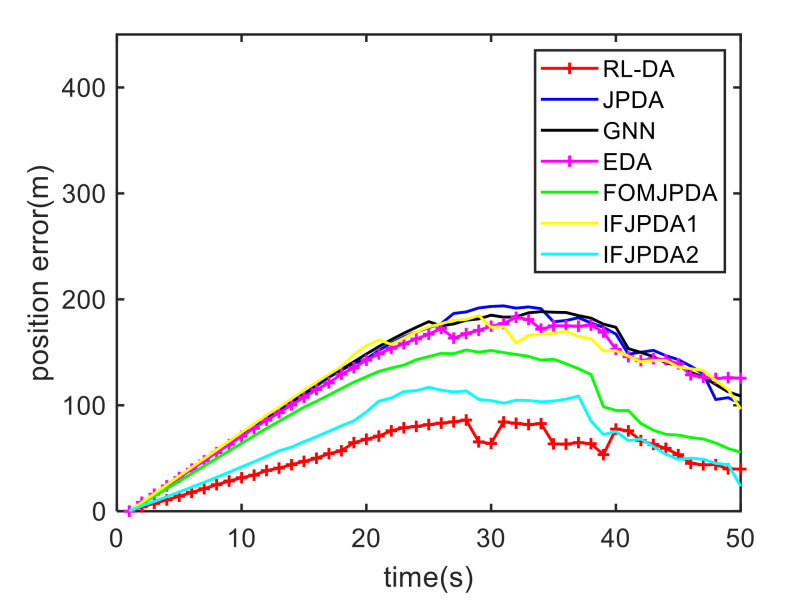
Comparison of the position errors for Target 2 in Case 2.

**Figure 27:**
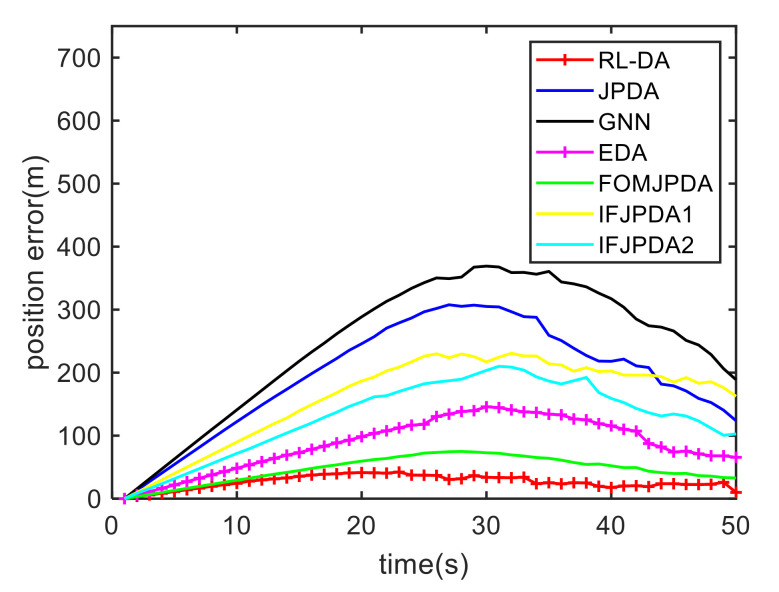
Comparison of the position errors for Target 3 in Case 2.

For Case 1, Figure 20 shows the trajectory estimation result of the proposed method. The true trajectories consist of three crossing tracks, and the estimated result of the proposed method has excellent performance. The mean position errors of the seven methods are illustrated in Figure 21, Figure 22 and Figure 23. The performance of the RL-JPDA method is better than the performance of all other methods, because the proposed method can acquire the motion characteristics of the reentry vehicle by training and online learning, which can improve the accuracy of data association. For the second case, Figure 24 shows the trajectory estimation result of the RL-JPDA method. The trajectory associated by RL-JPDA method still owns better performance. The mean position error of seven methods in Case 2 are illustrated in Figure 25, Figure 26 and Figure 27. Because of the proximity of the targets in Case 2, we can see that the position error of Case 2 is larger than that of Case 1. This is mainly due to the fact that close targets will increase the chance of error association, which make a decrease in performance for all methods. However, the results of EDA in Case 1 and Case 2 change slightly, which means the performance of EDA is not affected obviously by the change of distance between targets. The proposed method has great performance than other methods for solving the data association mission of close targets.

Moreover, the comparison results of the RMS errors and execution time are illustrated in Table 3 with the clutter rate $\lambda_{z}=10$ (for the realistic reentry vehicle tracking, the clutter rate cannot be too high). As shown in Table 3, because of the nonlinear variation caused by aerodynamic drag, the results of GNN and JPDA own poor performance. The RMS errors of RL-JPDA in Case 1 are 34.90 m, 32.69 m and 32.19 m, which proves that the proposed method still has great association effect for a nonlinear motion model. The performance of IF-JPDA2 is better than that of FOMJPDA and IF-JPDA1, but it is worse than the proposed method. The execution time of all methods is extended due to the frequent invocation of the objective dynamics function during the association process. However, the execution time of RL-JPDA is 1.30 s, and the execution time of JPDA is 2.82 s. These data indicate that the computational complexity of RL-JPDA method is lower than that of JPDA. Meanwhile, when the seven methods are used to solve the problem of close targets data association, the execution time of JPDA and IFJPDA2 is extended obviously, because close targets can increase the number of situations that measurement is assigned to multiple targets, which would significantly increase the number of joint event matrix in the two methods. However, the execution time of RL-JPDA is 1.32 s, and RMS errors of RL-JPDA are 45.01 m, 58.61 m and 28.41 m. These data indicate that the proposed method still has better performance.

In summary, from the above experimental results we can see that the combination of RL and JPDA can significantly improve the trajectory association performance, especially in the dense clutter environment. The structure of the JPDA method provides reliable association accuracy. Table 1, Table 2 and Table 3 show that the execution time of RL-JPDA is much less than that of JPDA. These data indicate that JPDA method has higher computational complexity, and the integration of reinforcement learning process into the traditional JPDA method facilitates a better handling of measurement clutters so as to achieve effective data association results. Meanwhile, the position information of measurements inside the tracking gate is also taken into full account. The motion characteristics of the targets are introduced as a constraint, which further improve the association performance of the proposed method.

### 4.4. Analysis of RL-JPDA Control Parameters

The value of training process parameter $K_{train}$ is set according to the situation that the initial segment of multiple target tracking is clutter free. If the length of training process is short, the accuracy of data association will be affected in the initial segment of multiple target tracking. Meanwhile, the clutter density of data association will increase gradually with the association process, so the training process should not be too long. The parameter setting of the change factor △ affects the performance of the RL. When the change factor △ approaches 1, the metric $D_{1,j}^{t}\left(k\right)$ will fluctuate dramatically if the RL action is switched. Furthermore, the change of $D_{1,j}^{t}\left(k\right)$ will be ignored when a small value of △ is given. The procedure parameter ν is set according to the motion characteristics of the target. The value of ν cannot be large due to the fact that there are errors in the dynamic model of the target. In addition, tracking gate is an important underlying support technology of the data association method. The value of tracking gate size ζ should be appropriate to contain as few clutters and interference as possible, which can ultimately improve the data association performance.

## 5. Conclusions

In this paper, a novel data association method based on reinforcement learning called RL-JPDA has been presented for solving multiple target tracking data association problems in the environment with dense clutters. The proposed method reconstructs the compute mode of joint association probabilities in JPDA by the method of reinforcement learning. The reinforcement learning is inserted to acquire the available information of measurements. The distribution of measurements is defined as states in RL and the estimated results are regarded as the evaluative signals. Particularly, the learning process of each target data is independent, which means that same distribution of measurements may have different association results for different targets due to the independent Q-table. In addition, the motion characteristics of the targets are developed to ensure the accuracy of the association results. Finally, the performance of the proposed method has been tested using six different methods in three scenarios, and these methods are compared in terms of error statistics and execution time. The results show that the RL-JPDA method is superior to the other six methods, and it can solve the data association problem effectively in the environment with dense clutters.

## Figures and Tables

**Table 1:** Root mean square (RMS) errors and execution time of the two cases.

Method	$\mathrm{Case}\;1\;(\lambda_{z}=20)$			$\mathrm{Case}\;2\;(\lambda_{z}=40)$		
	Time (s)	Target1 (m)	Target2 (m)	Time (s)	Target1 (m)	Target2 (m)
GNN	0.41	46.18	49.71	0.85	50.05	62.02
JPDA	1.93	40.65	43.40	\	\	\
EDA	0.38	27.15	27.78	0.42	39.68	33.51
FOMJPDA	0.42	34.42	27.67	0.59	38.37	35.54
IFJPDA1	0.64	21.39	30.47	0.87	37.49	38.09
IFJPDA2	0.70	25.74	20.27	1.34	33.91	31.73
RL-JPDA	0.46	16.74	17.21	0.63	24.90	26.60

**Table 2:** RMS errors and execution time of the two cases.

Method	$\mathrm{Case}\;1\;(\lambda_{z}=20)$				$\mathrm{Case}\;2\;(\lambda_{z}=40)$			
	Time (s)	Target1 (m)	Target2 (m)	Target3 (m)	Time (s)	Target1 (m)	Target2 (m)	Target3 (m)
GNN	0.70	181.82	99.57	147.61	4.35	202.35	285.72	191.61
JPDA	4.91	83.42	65.61	86.81	\	\	\	\
EDA	0.57	235.34	141.45	155.17	0.69	255.36	175.94	229.21
FOMJPDA	0.58	159.09	93.88	102.53	0.82	211.05	114.57	188.30
IFJPDA1	1.18	85.25	103.61	85.95	1.32	166.26	170.68	160.86
IFJPDA2	1.43	103.01	85.80	117.36	10.45	129.10	124.53	115.94
RL-JPDA	0.72	44.48	57.21	61.94	0.92	84.99	44.56	79.65

**Table 3:** RMS errors and execution time of the reentry vehicle example.

Method	$\mathrm{Case}\;1\;(\lambda_{z}=10)$				$\mathrm{Case}\;2\;(\lambda_{z}=10)$			
	Time (s)	Target1 (m)	Target2 (m)	Target3 (m)	Time (s)	Target1 (m)	Target2 (m)	Target3 (m)
GNN	1.32	144.65	190.73	229.48	1.54	175.49	139.41	268.45
JPDA	2.82	149.73	119.44	164.71	5.71	235.49	139.66	213.64
EDA	1.13	113.04	149.83	111.97	1.18	101.62	133.98	96.57
FOMJPDA	1.42	102.50	88.32	101.38	1.45	117.89	105.78	50.99
IFJPDA1	1.35	85.63	68.90	54.51	1.28	87.08	134.43	174.84
IFJPDA2	1.81	60.95	41.42	55.04	2.34	62.56	77.48	142.42
RL-JPDA	1.30	34.90	32.69	32.19	1.32	45.01	58.61	28.41
